# Changes in the characteristics of patients treated for brain metastases with repeat stereotactic radiotherapy (SRT): a retrospective study of 184 patients

**DOI:** 10.1186/s13014-023-02200-z

**Published:** 2023-01-30

**Authors:** L. Kuntz, C. Le Fèvre, D. Jarnet, A. Keller, P. Meyer, A. Thiery, H. Cebula, G. Noel, D. Antoni

**Affiliations:** 1 Department of Radiation Therapy, Institut de Cancérologie Strasbourg Europe (ICANS), 17 Rue Albert Calmette, 67200 Strasbourg, France; 2 Medical Physics Unit, Institut de Cancérologie Strasbourg Europe (ICANS), 17 Rue Albert Calmette, 67200 Strasbourg, France; 3 Medical Information Department, Institut de Cancérologie Strasbourg Europe (ICANS), 3 Rue de La Porte de L’Hôpital, 67065 Strasbourg Cedex, France; 4 Department of Neurosurgery, University Hospitals of Strasbourg, 1 Avenue Molière, 67200 Strasbourg, France

**Keywords:** Radiotherapy, Salvage radiation, Stereotactic radiosurgery, Brain metastases, Reirradiation, Repeated radiosurgery, Oligorecurrence

## Abstract

**Purpose:**

Brain metastases (BMs) are the leading cause of intracranial malignant neoplasms in adults. WHO, Karnofsky performance status (KPS), age, number of BMs, extracerebral progression (ECP), recursive partitioning analysis (RPA), diagnosis-specific graded prognostic assessment (Ds-GPA) are validated prognostic tools to help clinicians decide on treatment. No consensus exists for repeat stereotactic radiotherapy (SRT) for BMs. The aim of this study was to review the changes in patient characteristics treated with repeated SRTs.

**Methods and materials:**

The data of patients treated between 2010 and 2020 with at least two courses of SRT without previous whole brain radiotherapy (WBRT) were reviewed. Age, WHO, KPS, ECP, type of systemic treatment, number of BMs were recorded. RPA, Ds-GPA and brain metastasis velocity (BMV) were calculated.

**Results:**

184 patients were treated for 915 BMs and received two to six SRTs for local or distant brain recurrence. The median number of BMs treated per SRT was 1 (range: 1–6), for a median of 4 BMs treated during all sessions (range: 2–19). WHO, Ds-GPA and RPA were stable between each session of SRT, whereas KPS was significantly better in SRT1 than in the following SRT. The number of BMs was not significantly different between each SRT, but there was a tendency for more BM at SRT1 (*p* = 0.06). At SRT1, patients had largest BM and undergo more surgery than during the following SRT (*p* < 0.001). 6.5%, 37.5% and 56% of patients were classified as high, intermediate, and low BMV, respectively, at the last SRT session. There was almost perfect concordance between the BMV-grade calculated at the last SRT session and at SRT2 (r = 0.89; *p* < 0.001).

**Conclusion:**

Repeated SRT doesn't lead to a marked alteration in the general condition, KPS was maintained at over 70% for more than 95% of patients during all SRTs. Long survival can be expected, especially in low-grade BMV patients. WBRT shouldn't be aborted, especially for patients developing more than twelve BMs annually.

## Purpose

Brain metastases (BMs) are the most common intracranial malignant neoplasms in adults [1]. From 10 to 40% of oncologic patients will develop BMs among their oncologic courses. Stereotactic radiation therapy (SRT) has become the treatment of choice in the management of new or recurrent BM. In contrast to tumoral cells, few systemic treatments cross the blood–brain barrier, explaining the low efficacy of chemotherapies/targeted treatments to prevent BM development and leading to the consideration of the brain as a sanctuary site [2]. Kuntz et al. showed that 20–40% of patients will require salvage treatment after an initial SRT [3]. Different treatment options are available for recurrent cranial metastatic disease, including repeated SRT, whole-brain radiotherapy (WBRT), surgery, systemic therapy, and supportive care. The main benefits of repeated SRT are to delay WBRT, which causes cognitive toxicities and a reduction in quality of life [4–6]. The factors influencing the treatment choice and its modality are numerous, including the number, location, and size of the BM, the patient's general condition, histologic type of primary cancer, extracranial status and life expectancy [7, 8].

Our monocentric retrospective study reports patients who underwent repeated SRT without upfront or intercalated WBRT, focusing on the evolution of the patients' characteristics over time and during every SRT. The aim is to review the changes in patient characteristics treated with repeated SRT over time.

## Methods and materials

### Patients and treatment modalities

We queried our institutional database to obtain the list of patients who received two or more SRT sessions for cerebral or local recurrences for a single or multiple BMs. No minimal follow-up duration was needed after SRT2.

Patients who had previous WBRT or WBRT after only one SRT session were excluded. We identified 184 patients treated for 915 BMs between January 2010 and June 2020. SRT was mostly delivered in one fraction of 20 Gy (n = 235; 26%) or in three fractions of 11 Gy every 2 days (n = 659; 72%) on the 70% isodose line [9]. Treatments were delivered using volumetric modulated arc therapy (VMAT) or dynamic conformal arc treatment (DCA). Each SRT was carried out under cover of corticosteroid therapy, with a decreasing dose over a fortnight. If the patient was receiving intermittent systemic treatment, SRT was ideally performed during free intervals to avoid stopping it. If the patient was receiving continuous systemic treatment, stop of treatment was done for some drugs within 48–72 h before the first day of irradiation, and then resumed the day after irradiation.

For each patient and at each SRT session, age, WHO, Karnofsky performance status (KPS), ECP, administration and type of systemic treatment, number of BMs, resected BMs and recurrent BMs were recorded. We were then able to calculate the recursive partitioning analysis (RPA) and diagnosis-specific graded prognostic assessment (Ds-GPA) for each patient and at each SRT session according to the histology of the primary cancer [10].

### Brain metastasis velocity (BMV)

The final brain metastasis velocity (BMV) and BMV at each session starting from SRT2 were calculated for each patient according to Farris et al., such as Yamamoto et al., who confirmed the validity of BMV in predicting overall survival (OS) after the second SRT but also after the third and after the fourth [11, 12]. Final BMV was calculated by dividing all the new BMs starting from SRT1 by the time in year between SRT1 and the last SRT session. BMV at each SRT session was calculated by dividing all the new BMs starting from SRT2, SRT3 and SRT4 by the time in year between SRT1 and SRT2, SRT2 and SRT3, SRT3 and SRT4. BMV was classified into low-, intermediate-, or high-risk groups if the number of new BMs was strictly < 4, 4–13 and > 13, respectively.

### Local recurrence

Follow-up MRI was performed every 3–6 months after SRT to diagnose LR, cerebral recurrence, radionecrosis (RN), stable disease, partial response, or complete response. A new contrast enhancement outside the previously treated BM was categorized as a distant CR. A contrast enhancement inside the previously treated BM suggested an LR. Confirmation of LR was made by surgery or complementary examination, such as 18-FDG-PET-CT, F-DOPA-PET-CT, or a new MRI performed in a shorter interval. Differential diagnosis between RN and LR is complicated, even more so as in 50% of cases the necrosis is tumorous [13, 14]. Clinical arguments in favor of LR were the presence of neurological symptoms or no response to corticosteroid therapy. MRI arguments in favor of LR were a lesion quotient (ratio of maximum areas of T2 to T1) greater than 0.3 or 0.6 [15–18]. In nuclear medicine, radiotracers commonly used to differentiate radionecrosis and LR are 18-fluorodeoxyglucose (FGD). 18-FDG-PET-CT finds hypermetabolism and late retention of the radiotracer in cases of tumor recurrence [13, 14, 19].

### Statistical analysis

The quantitative variables were described using standard position and dispersion statistics, namely, the mean, median, variance, minimum, maximum and quantiles. The qualitative data were described along with the numbers and proportions of each modality. Cumulative proportions were also calculated for variables with more than two modalities. Gaussian distributions of the quantitative variables were assessed using the Shapiro–Wilk test. If the conditions were met, the relationship between two quantitative variables was assessed using Pearson's linear correlation test. Otherwise, a Spearman correlation test was performed. For the comparison of a quantitative variable between several subgroups, an analysis of variance was used. For the comparison of a quantitative variable between several subgroups, an analysis of variance or the Kruskal and Wallis test were used, again according to the assumptions of use of each of these tests. Finally, for the crossing between several qualitative variables, the parametric Chi^2^ test was used if the conditions of application allowed it. If this was not the case, the exact Fisher test was carried out. The alpha risk was set at 5% for all analyses. All analyses were performed using R software version 3.1, R Development Core Team (2008, R Foundation for Statistical Computing, Vienna, Austria) and GMRC Shiny Stat (2017).

## Results

### Patients’ characteristics

**Fig. 1:**
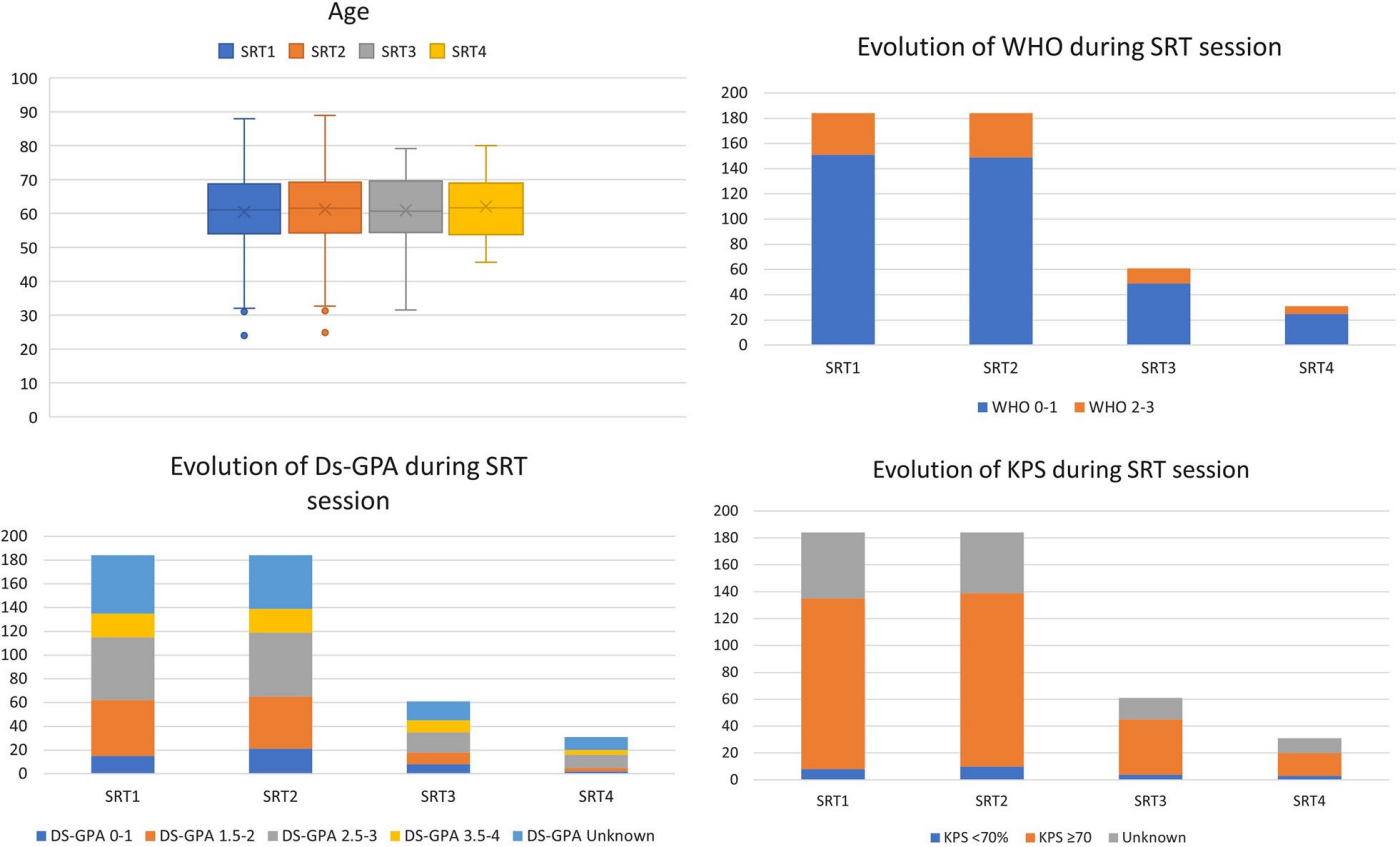
Evolution of patient characteristics at each session of SRT. *BM* brain metastases, *DS-GPA* diagnosis-specific graded prognostic assessment, *ECP* extracerebral progression, *KPS* Karnofsky performance score, *WHO* performance status, *RPA* recursive partitioning analysis, *SRT* stereotactic radiotherapy, *SRT1* first session of stereotactic radiotherapy, *SRT2* second session of stereotactic radiotherapy, *SRT3* third session of stereotactic radiotherapy, *SRT4* forth session of stereotactic radiotherapy

A total of 184 patients were enrolled, 56% were followed for lung cancer, 13% for breast cancer, 13% for melanoma, 8.7% for digestive cancer, 4.3% for kidney cancer and 4.9% for other cancers. Table 1 shows the patients and primitive tumor characteristics. One hundred and twenty-two patients (66.3%) received two sessions of SRT, 40 (21.7%) received three sessions, 14 (7.6%) received four sessions, 7 (3.8%) received five sessions, and one (0.5%) received six sessions, totaling 461 administered treatments and 915 treated BM. Twenty-six percent of BMs were irradiated with a monofractionated, 72% of BMs were irradiated with a trifractionated regimen, and the last 2% of BMs were irradiated with other hypofractionated schedules. A mean of two BM was treated per SRT (range: 1–6; 95% CI 1.88–2.12), for a total average of 5 BMs treated during all sessions (range: 2–19; 95% CI 4.52–5.44). All characteristics are shown in Fig. 1.

**Table 1:** Patients and primitive tumor characteristics (n = 184)

Characteristics	Number	Percentage
*Sex*		
Male	91	49.5
Female	93	50.5
*Age at diagnosis of cancer*		
Median (range)	58 (21–87)	
≤ 65 yo	135	73
> 65 yo	49	27
*Age at diagnosis of brain metastasis*		
Median (range)	61 (24–88)	
≤ 65 yo	124	67
> 65 yo	60	33
Primitive cancer		
Lung	103	56.0
Adenocarcinoma	69	67
Epidermoid	19	18.4
Small cell	4	3.9
Undifferentiated	4	3.9
Other	7	6.8
Breast	24	13.0
Luminal A	5	20.8
Luminal B	5	20.8
Her2+	11	45.8
Triple negative	3	12.5
Melanoma	24	13.0
Kidney	8	4.3
Digestive	16	8.7
Other	9	4.9
*Initial tumor stage*		
1	23	12.5
2	47	25.5
3	38	20.7
4	30	16.3
Unknown	46	25.0
*Initial node stage*		
0	37	20.1
1	23	12.5
2	49	26.6
3	26	14.1
Unknown	49	26.6
*Initial metastasis stage*		
0	81	44.0
1	90	48.9
Unknown	13	7.1

### Overall condition

The median WHO was 1 ± 0.79 at SRT1, 1 ± 0.75 at SRT2, 1 ± 0.68 at STR3 and 1 ± 0.65 at SRT4 and more (SRT4-5–6), with *p* = 0.39, *p* = 0.07 and *p* = 0.64, respectively, before and after each consecutive session of SRT. The median KPS was 90% ± 16% at SRT1, 80% ± 14% at SRT2, 90% ± 13% at STR3 and 90% ± 16% at SRT4-5-6, with *p* = 0.02, *p* = 0.16 and *p* = 0.07, respectively, between each consecutive session. The KPS at SRT1 was significantly different from the KPS at SRT3 (*p* = 0.02) but not from the KPS at SRT4 (*p* = 0.32). The median Ds-GPA was 2.5 ± 0.92 at SRT1, 2.5 ± 1.03 at SRT2, 2.5 ± 1.09 at STR3 and 3 ± 0.65 at SRT4-5-6, with *p* = 0.52, *p* = 0.89 and *p* = 0.5, respectively, between each consecutive session. The median RPA was 2 ± 0.44 at SRT1, 2 ± 0.51 at SRT2, 2 ± 0.53 at STR3 and 2 ± 0.41 at SRT4-5-6, with *p* < 0.37, *p* = 0.09 and *p* = 1, respectively, between each consecutive session of SRT.

### Oncological status

**Fig. 2:**
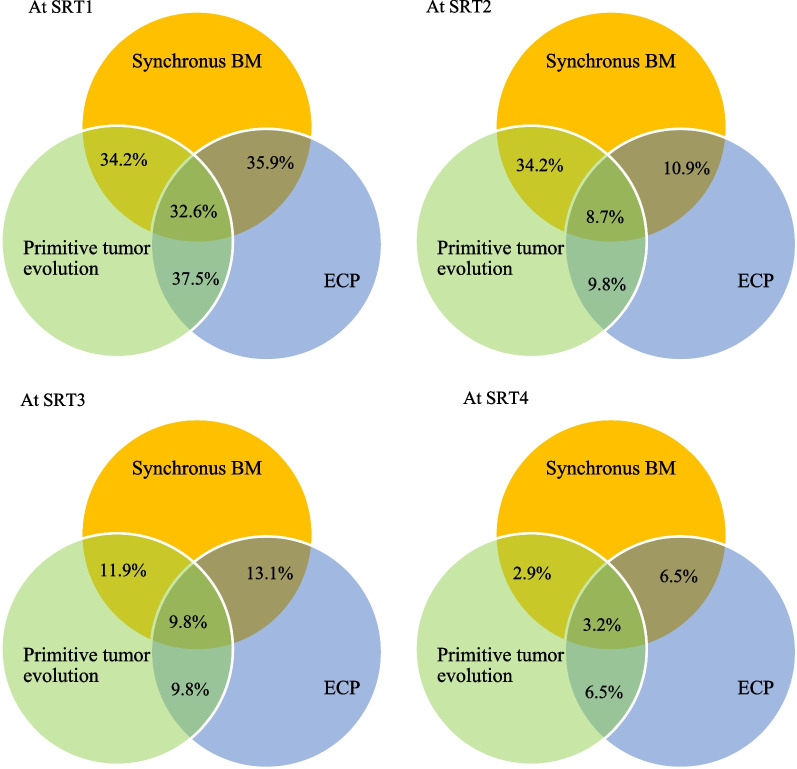
Evolution of oncological status at each SRT. *BM* brain metastases, *ECP* extracerebral progression, *SRT1* first session of stereotactic radiotherapy, *SRT2* second session of stereotactic radiotherapy, *SRT3* third session of stereotactic radiotherapy, *SRT4* fourth session of stereotactic radiotherapy

Sixty-four percent of patients were diagnosed with ECP at SRT1, and 37.5% of patients were diagnosed with brain metastatic disease at initial diagnosis. Twenty-eight percent of patients have ECP at SRT1 during follow-up of cancer. Synchronous BM diagnosis with primitive tumors was observed in 53.4% of lung cancer cases, 37.5% of kidney cancer cases, 20.8% of melanoma cases and 18.8% of digestive cancer cases but not in any breast cancer cases. Figure 2 represents the evolution of oncological status at each SRT. There was no statistically significant difference between patients with synchronous or metachronous brain metastatic at SRT1 and ECP patients at SRT1 who received more than 3 SRT sessions (*p* = 0.49 and *p* = 0.8, respectively) or more than 4 SRT sessions (*p* = 0.41 and *p* = 0.64, respectively). There was no significant difference in ECP between SRT1 and noninitial brain metastatic patients who received more than 3 SRT sessions (*p* = 0.31) or more than 4 SRTs (*p* = 0.29). ECP was present for 64% of patients at SRT1 compared to 30% at SRT2 (*p* < 0.001), 23% at SRT3 (*p* = 1) and 23% at SRT4-5-6 (*p* = 1) in before-after analysis between each succeeding session. By comparing patients with no extracerebral synchronous BM with others at SRT1, there was no significant difference between ECP between SRT1 and SRT2 (*p* = 0.73). Patients who were extracranial metastasis (ECM)-free at cancer diagnosis and ECP-free at SRT1 and SRT2 were not significantly more likely to receive three or more SRT sessions than those with ECM at diagnosis (*p* = 0.58) and/or ECP at SRT1 and SRT2 (*p* = 0.8 and *p* = 0.27, respectively).

### Systemic therapy

Seventy percent, 68%, 77%, and 61% of patients were on systemic therapy at SRT1, SRT2, SRT3 and SRT4-5-6, respectively. Patients who had uncontrolled primary tumors at diagnosis of brain metastases and ECM were more likely to receive systemic therapy at SRT1 (*p* = 0.04 and *p* < 0.01, respectively). Patients who had ECM at SRT2 and/or SRT3 were more likely to receive systemic therapies than patients with no ECM (*p* < 0.001 and < 0.001, respectively). Patients who had ECP at SRT2 and/or SRT3 were more likely to receive systemic therapies than patients who were ECP-free (*p* < 0.001 and *p* = 0.03, respectively). Systemic therapies were divided into 5 classes: chemotherapy (CT), targeted therapy (TT), immunotherapy (IT), hormonotherapy (HT), and combination therapy. In the before-after comparison between each consecutive session, there was no significant difference in the use of the different classes of treatments between SRT1 and SRT2 or between SRT2 and SRT3 (*p* = 0.8 and *p* = 0.8, respectively). Fifty-nine percent, 50%, 43% and 26% of patients received CT at SRT1, SRT2, SRT3 and SRT4 and above, respectively, and there was no significant difference between each consecutive session in the before-after comparison (*p* = 0.11, *p* = 1, and *p* = 0.48, respectively). The use of CT was statistically associated with progression of the primary tumor site (*p* < 0.001 at SRT1 and *p* < 0.001 at SRT2). A total of 11%, 15%, 15% and 3% of patients received IT at SRT1, SRT2, SRT3 and SRT4 and above, respectively, and there was no significant difference between each session (*p* = 0.45, *p* = 0.48 and *p* = 1, respectively). Twenty-three percent, 22%, 32% and 29% of patients received TT at SRT1, SRT2, SRT3 and SRT4 and above, respectively, and there was no significant difference between each session (*p* = 0.72, *p* = 0.68 and *p* = 1, respectively).

Patients treated with TT at SRT1 and/or SRT2 tended to be more likely to receive more than three SRT sessions than other patients (*p* = 0.16 and *p* = 0.15, respectively). TT was statistically associated with primitive tumoral control at SRT1 (*p* < 0.001) and SRT2 (*p* = 0.01) and ECP at SRT1 (*p* = 0.05). TT tended to be statistically associated with primitive tumoral control at SRT3 (*p* = 0.11). TT was not statistically associated with primitive tumoral control at SRT4 (*p* = 0.59) or ECP at SRT2 (*p* = 0.50). The following associations were not calculable due to the small number of patients and events.

### Interval time between consecutive SRT

The median delay between each consecutive SRT session was 9.2 months (range 0.9–69.9; 95% CI 7.9–10.6) between SRT1 and SRT2, 5.3 months (range 1.1–61.5; 95% CI 6.1–11.5) between SRT2 and SRT3, 7.8 months (range 2.7–54.8; 95% CI 2.9–19.1) between SRT3 and SRT4 and 7.2 months (range 3.1–52.5; 95% CI 5.3–15.1) between SRT4 and the following SRT sessions. There was no significant difference in the before-after analysis between each consecutive time interval (*p* = 0.57, *p* = 0.66 and *p* = 0.11, respectively).

The median time interval between SRT1 and SRT2 was 8.1 months (95% CI 7.7–11.0) for patients with no systemic treatment at SRT2, compared with 7.0 months (95% CI 8.4–16.4) for patients with maintenance treatment and 5.3 months (95% CI 5.3–8.8) for patients with active treatment (*p* < 0.001). There was also a trend towards a statistical association for the time interval between SRT2 and SRT3 and maintenance treatment at SRT3 (*p* = 0.09). There was no statistical association between the time interval between consecutive SRT and ECP at SRT2, SRT3 and SRT4 or higher (*p* = 0.24, *p* = 0.33 and *p* = 0.65, respectively).

### Cerebral status

The median number of BMs was 1.5 (95% CI 1.0–6.0) at SRT1, 1 (95% CI 1.0–5.0) at SRT2, 1 (95% CI 1.0–4.0) at SRT3 and 1 (95% CI 1.4–2.2) at SRT4-5-6. Among the 915 BMs, 96 were operated on before SRT (10%). Seventy patients (38%) underwent surgery for one or two BMs at SRT1, 18 (10%) at SRT2, 4 (7%) at SRT3 and 2 at SRT4-5-6. The number of BMs between each SRT was not significantly different between each SRT, but there was a tendency for more metastases in SRT1 (*p* = 0.06). The number of BMs at SRT1 tended to be associated with control of the primitive tumoral site (*p* = 0.14), ECM at cancer diagnosis (*p* = 0.1) and ECP at SRT1 (*p* = 0.24) but was not associated with the use of a third SRT session (*p* = 0.95). Breast cancer, lung cancer and melanoma provided more BMs at SRT1 (*p* < 0.001) and more total BMs (*p* = 0.05) than other primitive cancers.

The median GTVs of each BM at SRT1, SRT2, SRT3 and SRT4-5-6 were 0.4 mL (95% CI 1.84–2.94), 0.4 mL (95% CI 2.67–4.77), 0.25 mL (95% CI 1.42–4.43), and 0.35 mL (95% CI 1.16–4.16), respectively. Total GTV at SRT1 was statistically higher than GTV at SRT2 (*p* < 0.001), but this difference was not found between SRT2 and SRT3 (*p* = 0.34) or between SRT3 and SRT4-5-6 (*p* = 0.62). A high GTV at SRT1 was not statistically associated with synchronous brain metastases at diagnosis (*p* = 0.43).

### Local recurrences (LR)

Twenty-seven patients (15%) were reirradiated for one or more local relapses at SRT2, 10 (17%) at SRT3 and 8 (26%) at SRT4 and above. Among the 93 BMs with local relapse, 63.4% recurred after the first session, 26.8% after the second session, 4.3% after the third session, 4.3% after the fourth session, and 1% after the fifth session (*p* < 0.001). LR was confirmed by MRI only for 66 BMs (71.0%), by nuclear medicine only for 18 BMs (19.4%), by both for 2 BMs (2.2%) and by surgery for 6 BMs (6.5%). The median interval time between SRT and local relapse was 9.2 months (95% CI 10.6–18.0) for BMs treated at SRT1 and 7.5 months (95% CI 7.1–13.6) for BMs treated at SRT2 and more (*p* = 0.28). Fifty-one BMs were reirradiated after local recurrence, 56.8% were reirradiated at SRT2, 27.5% were reirradiated at SRT3, and 15.7% were reirradiated at SRT4 and above (*p* < 0.001).

### BMV grade and patient outcome

At SRT2, 8.1%, 33.7% and 58.2% of patients were classified as having high, intermediate, and low BMV, respectively; at SRT3, 7.6%, 36.4% and 56%, respectively; and at the last SRT session, 6.5%, 37.5% and 56%, respectively. There was almost perfect concordance between the BMV grade calculated at the last SRT session and that calculated at SRT2 (r = 0.89; *p* < 0.001). Salvage WBRT was used in 18.5% (34) of patients after a median time of 9.8 months (95% CI 8.6–15.9) after SRT1 and 3.9 months (95% CI 3.5–5.2) after the last SRT. This WBRT was used after SRT2, SRT3 and SRT4-5-6 in 24.4%, 7.7% and 4.5% of patients, respectively. Among patients with high BMV grade, 60% received 2 SRT sessions without WBRT, 20% received 3 SRT sessions or more without WBRT, and 20% ultimately received WBRT. Among patients with intermediate BMV grade, 54.8% received only 2 SRT sessions without WBRT, 16.1% received 3 SRT sessions or more without WBRT, and 29% ultimately received WBRT. Among patients with low BMV grade, 46.7% received only 2 SRT sessions without WBRT, 41.1% received 3 SRT sessions or more without WBRT, and 12.1% ultimately received WBRT (*p* = 0.02). The median interval time between SRT1 and WBRT was statistically longer for low-BMV patients than for other patients (*p* < 0.001).

### Distant and local brain failure

**Fig. 3:**
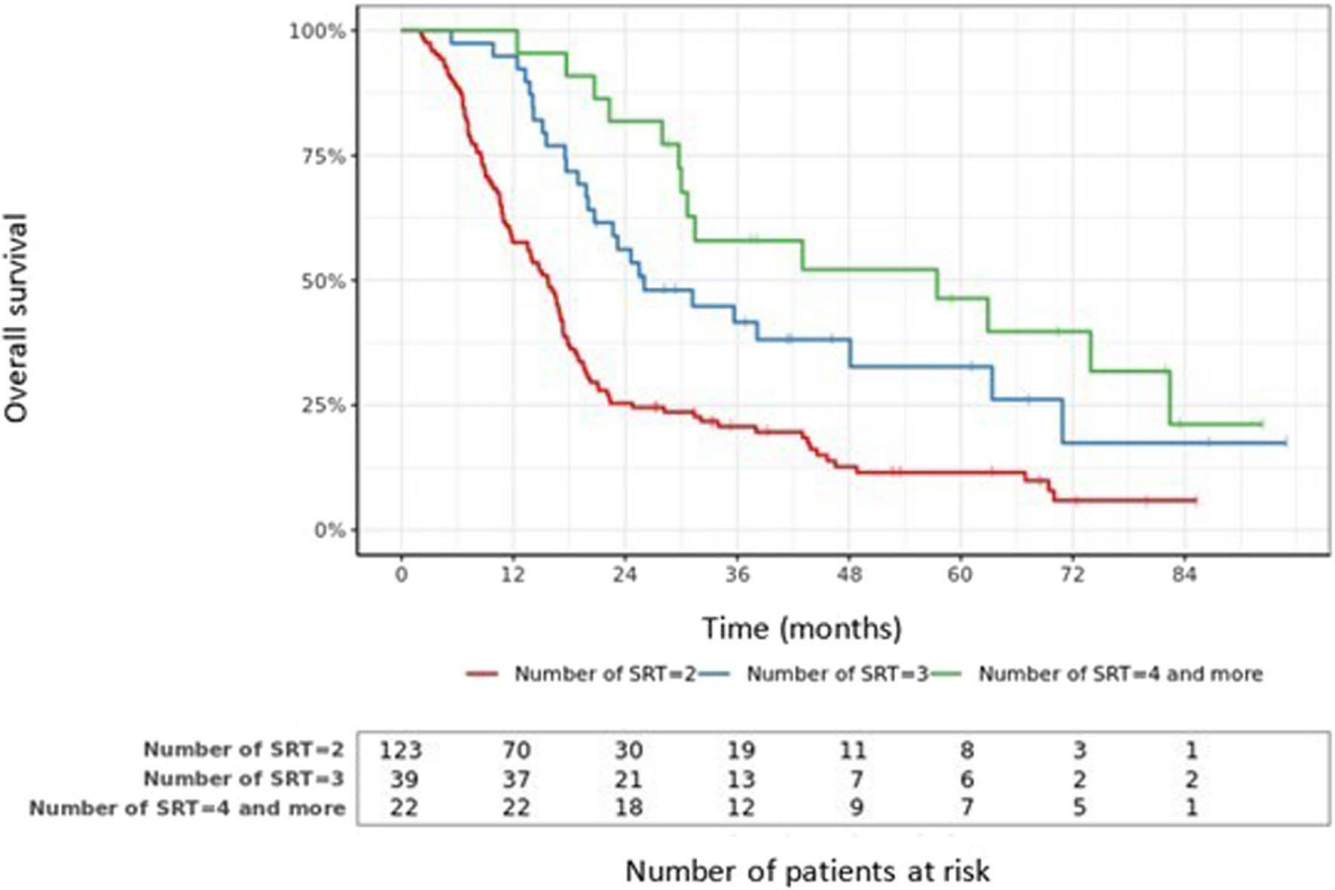
Overall survival in function of the number of SRT sessions

**Fig. 4:**
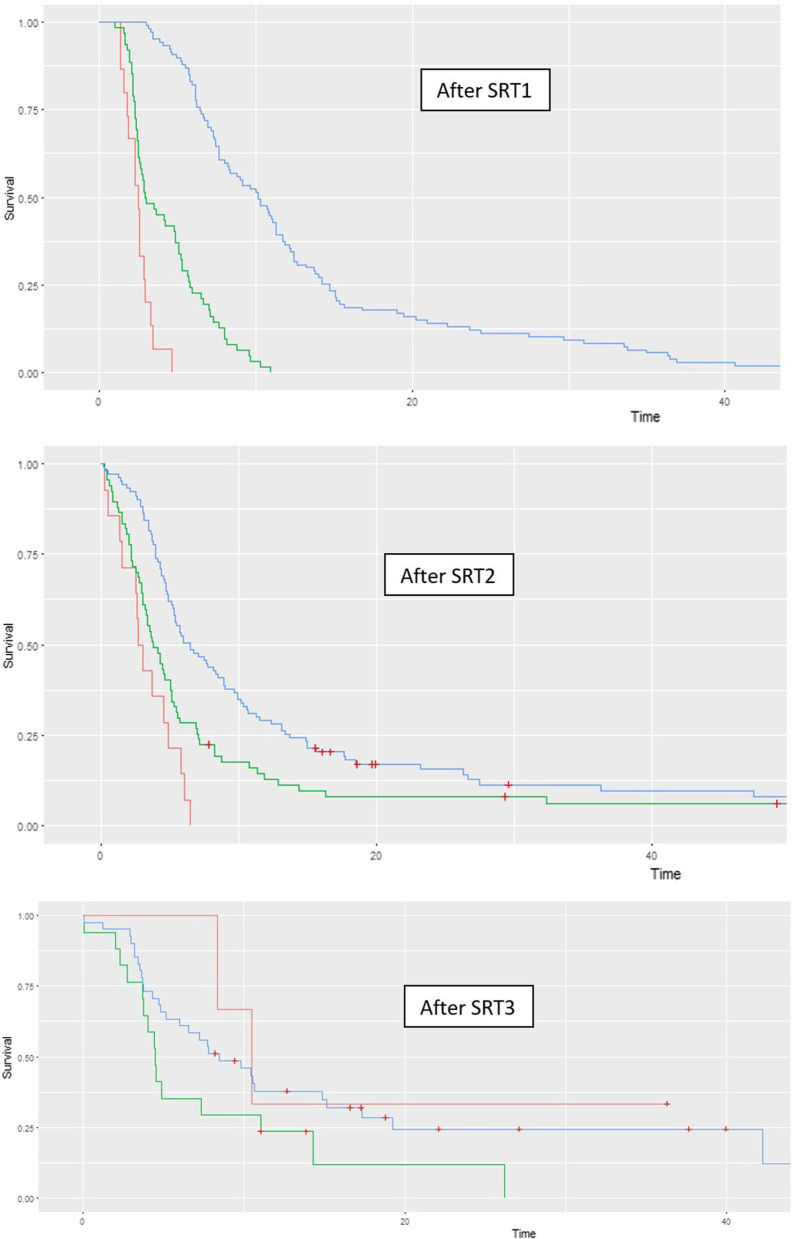
Survival without cerebral recurrence in terms of BMV grade (time in months). *SRT1* first session of stereotactic radiotherapy, *SRT2* second session of stereotactic radiotherapy, *SRT3* third session of stereotactic radiotherapy, *SRT4* forth session of stereotactic radiotherapy. Blue: Low BMV grade; Green: Intermediate BMV grade; Red: High BMV grade

The median follow-up time of the whole population was 18.4 months after SRT1 (range: 2–95). At this time point, 20.1% patients were still alive. The median OS was 18.6 months (95% CI 17.0–21.1). The six-, 12- and 24-month OS rates were 91% (95% CI 88–96), 70% (95% CI 64–77), and 38% (95% CI 32–45), respectively. Figure 3 shows overall survival in function of the number of SRT sessions. Distant Brain Failure (DBF) was defined as a new SRT session or WBRT. Median DFB was 6.8 months (95% CI 8.4–10.9). The median DFB after SRT1, SRT2, SRT3 and SRT4 and more were 6.6 (95% CI 7.9–10.6), 5.1 (95% CI 7.7–11.6), 6.7 (95% CI 8.3–14.1) and 7.7 months (6.1–18.9), respectively. Figure 4 shows survival without DBF after SRT1, SRT2 and SRT3 as a function of BMV grade. Three-, 6-, 12- and 24-months BM local control were 99.3%, 96.3%, 90.1% and 85.8%, respectively. DBR-RPA score was strongly associated with overall survival after first DBR [20]. Median OS after SRT2 among patients DBR-RPA-I, II and III were respectively, 11.6 months (IC95% 7.5–16.3), 13.4 months (IC95% 10.7–25.0) and 3.9 months (IC95% 2.5–8.1) (*p* < 0.001).

## Discussion

Patients diagnosed with a limited number of BMs may benefit from repeated SRT. Because the brain is not very accessible to systemic therapies, brain relapse is frequent [20]. Indeed, approximately 50% of patients treated for an initial SRT develop new BM 1 year after the first irradiation [21]. There are few data concerning repeated SRT of BM, and no study has assessed the evolution of the clinical characteristics of patients during the different treatment sequences. This retrospective study investigated a large series of patients with de novo or recurrent BMs treated with repeated SRT. Moreover, because our study is monocentric, this gives it a certain homogeneity, with notably 98% of the BMs who were irradiated in mono- or trifractionated schemes. A large amount of data was collected, and although some data were not calculable, there was little missing information.

Patients’ initial demographic characteristics are comparable with those of other series in the literature that study repeated courses of SRT [22–28].

Our study suggests that the KPS and the characteristics of BMs at the SRT1 are significantly different from those at SRT2. At the subsequent SRT, the KPS and the characteristics of the BMs remained statistically comparable. Indeed, we showed that the KPS in SRT1 was statically lower than that in SRT2; on the other hand, the WHO was stable during the consecutive SRT. This can be explained by the fact that most of the patients receiving SRT are in good general condition, classified as WHO grade 0 or 1. The KPS is a finer score, and good general condition patients will be classified from 100% KPS to 70%, which makes the difference easier to show. Kotecha et al. [23] studied 59 patients who underwent at least three SRT sessions for a total of 765 BMs. The median KPS at the last visit was 70%, and only 41% of patients had a deterioration in KPS after a median delay of 16 months.

With a median RPA of 2 and a median Ds-GPA of 2.5 without a significant difference between each consecutive session, RPA and Ds-GPA can be considered stable during repeated SRT. Indeed, the median RPA was 2 at all sessions without a significant difference between each consecutive session, and the median Ds-GPA was 2.5 at all sessions without a significant difference between each consecutive session. RPA and Ds-GPA are validated prognostic scores for OS in patients with BMs [29–31]. Ds-GPA is the only validated overall survival prognostic score that considers histological type. Ds-GPA has only been validated for the initial diagnosis of brain metastases, and to our knowledge, no prognostic score has been validated for the management of local or distant recurrence of BM. Yamamoto et al. [32] studied five prognostic indices, including RPA, GPA and other brain-specific scores, among 804 patients who underwent repeat SRT. They found a significant difference between RPA at SRT1 and RPA at SRT2 (*p* < 0.001), but their analysis showed that RPA does not adequately reflect the changes in patient conditions. In fact, 95% of their cohort had the same RPA score between SRT1 and SRT2. We found comparable results, with 75% of patients having a stable RPA between SRT1 and SRT2, 15% having a greater RPA and 10% having a lower RPA. Most of the stable RPA (89%) patients were classified as RPA II.

Base on two major prognostic factors, Zindler et al. [20] developed DBR-RPA prognostic score. Our study shows survival results comparable with the outcomes of Zindler et al. with notably a survival of more than 10 months after the first cerebral recurrence in patients in the favorable group. Although the BMs characteristics are comparable to those of other series in the literature that study repeated courses of SRT [22–28], none of these studies investigated their evolution over time. Based on these findings, BMs treated in SRT1 were larger, more numerous and more frequently operated on than BMs in subsequent SRTs. More than half of synchronous brain metastatic patients had lung cancer, followed by kidney cancer and melanoma. Shibahara et al. studied 471 patients treated for BMs of any primary origin; 20% were included in the precocious group, 16% in the synchronous group and 64% in the metachronous group [33]. The diameter of BMs was significantly higher in the precocious-group, followed by the metachronous-group and synchronous-group (*p* < 0.001). A systematic and close brain follow-up could decrease the size and number of metachronous BMs and avoid the loss of a treatment opportunity. For cancers for which brain monitoring is not systematic, risk prediction models can be used to justify the prescription of brain imaging in a given patient [34, 35].

A few studies have looked at prognostic factors during salvage treatment, and the main factors found were the number of BMs, time interval between SRT sessions, KPS, ECP status and, more recently, the BMV grade [11, 25, 36]. We observed an almost perfect correlation between the BMV grade calculated at the first cerebral relapse and the BMV grade calculated at the last treatment session. Among the 6% of patients who had changes in BMV grade, more than half of the group changed because they were close to 4 of 13 BMs per year at SRT2, and they changed groups because of a small change in BMV.

We found no link between the use of systemic therapy and overall survival or multiple sessions as other studies in the literature. Nevertheless, IT and TT have revolutionized patient management and outcomes, particularly in lung cancer, melanoma, triple-negative or HER2-positive breast cancer [37–42]. Further studies including more patients are needed to explain the decrease in the rate of spread of new tumor cells and new distant BMs with repeated courses of SRT in patients on immunotherapy or targeted therapy.

Selection of the best candidates for repeated SRT and avoiding WBRT as much as possible is essential, especially for patients who will benefit from 3 SRTs or more. Patients who have reached the third SRT session remain clinically, oncologically and neurologically stable at each treatment session, whereas their condition changes between SRT1 and SRT2. Although our study focuses on a specific and well-selected population, brain reirradiation under stereotactic conditions is becoming increasingly common.

## Conclusion

In this retrospective study including 184 patients treated with repeated postoperative stereotactic radiosurgery or SRT for cerebral or locally recurrent BM, we studied the evolution of patient characteristics over time. Patient characteristics seem to be different between SRT1 and SRT2, and then stable during all repeated SRT sessions, especially KPS, ECP and BM volume, number, and surgical management. However, the KPS was maintained over 70% for more than 95% of patients during all SRTs. Repeated SRT is a feasible treatment modality, and further studies are needed to define the optimal evolution of patients with recurrent brain metastases.

## Data Availability

Not applicable.

## Abbreviations

BM: Brain metastasis
BMV: Brain metastases velocity
CT: Chemotherapy
DCA: Dynamic conformal arc treatment
DS-GPA: Diagnosis-specific graded prognostic assessment
ECM: Extracranial metastasis
ECP: Extracerebral progression
GTV: Gross tumor volume
HT: Hormonotherapy
IT: Immunotherapy
KPS: Karnofsky performance score
LR: Local recurrence
OS: Overall survival
RPA: Recursive partitioning analysis
SRT: Stereotactic radiotherapy
SRT1: First session of stereotactic radiotherapy
SRT2: Second session of stereotactic radiotherapy
SRT3: Third session of stereotactic radiotherapy
SRT4: Fourth session of stereotactic radiotherapy
TT: Targeted therapy
VMAT: Volumetric modulated arc therapy
WBRT: Whole-brain radiotherapy
WHO: WHO performance status

## References

[1] Nayak L, Lee EQ, Wen PY (2012). Epidemiology of brain metastases. Curr Oncol Rep.

[2] Puhalla S, Elmquist W, Freyer D, Kleinberg L, Adkins C, Lockman P (2015). Unsanctifying the sanctuary: challenges and opportunities with brain metastases. Neuro Oncol.

[3] Kuntz L, Noel G. Repeated irradiation of brain metastases under stereotactic conditions: a review of the literature. Cancer Radiother. 2021. 10.1016/j.canrad.2020.08.050 33431294

[4] Brown PD, Jaeckle K, Ballman KV, Farace E, Cerhan JH, Anderson SK (2016). Effect of radiosurgery alone vs radiosurgery with whole brain radiation therapy on cognitive function in patients with 1 to 3 brain metastases: a randomized clinical trial. JAMA.

[5] Aoyama H, Tago M, Kato N, Toyoda T, Kenjyo M, Hirota S (2007). Neurocognitive function of patients with brain metastasis who received either whole brain radiotherapy plus stereotactic radiosurgery or radiosurgery alone. Int J Radiat Oncol Biol Phys.

[6] Bunevicius A, Lavezzo K, Shabo L, McClure J, Sheehan JP (2020). Quality-of-life trajectories after stereotactic radiosurgery for brain metastases. J Neurosurg.

[7] Chidambaram S, Pannullo SC, Schwartz TH, Wernicke AG (2019). Reirradiation of recurrent brain metastases: where do we stand?. World Neurosurg.

[8] Ammirati M, Cobbs CS, Linskey ME, Paleologos NA, Ryken TC, Burri SH (2010). The role of retreatment in the management of recurrent/progressive brain metastases: a systematic review and evidence-based clinical practice guideline. J Neurooncol.

[9] Latorzeff I, Antoni D, Gaudaire-Josset S, Feuvret L, Tallet-Richard A, Truc G (2016). Radiothérapie des métastases cérébrales. Cancer/Radiothérapie.

[10] Likhacheva A, Pinnix CC, Parikh N, Allen PK, Guha-Thakurta N, McAleer M (2012). Validation of recursive partitioning analysis and diagnosis-specific graded prognostic assessment in patients treated initially with radiosurgery alone. J Neurosurg.

[11] Farris M, McTyre ER, Cramer CK, Hughes R, Randolph DM, Ayala-Peacock DN (2017). Brain metastasis velocity: a novel prognostic metric predictive of overall survival and freedom from whole-brain radiation therapy after distant brain failure following upfront radiosurgery alone. Int J Radiat Oncol Biol Phys.

[12] Yamamoto M, Aiyama H, Koiso T, Watanabe S, Kawabe T, Sato Y (2019). Validity of a recently proposed prognostic grading index, brain metastasis velocity, for patients with brain metastasis undergoing multiple radiosurgical procedures. Int J Radiat Oncol Biol Phys.

[13] Wang B, Zhang Y, Zhao B, Zhao P, Ge M, Gao M (2018). Postcontrast T1 mapping for differential diagnosis of recurrence and radionecrosis after gamma knife radiosurgery for brain metastasis. AJNR Am J Neuroradiol.

[14] Delmaire C, Savatovsky J, Boulanger T, Dhermain F, Le Rhun E, Météllus P (2015). Brain metastases imaging. Cancer Radiother.

[15] Menoux I, Armspach J-P, Noël G, Antoni D (2016). Imaging methods used in the differential diagnosis between brain tumour relapse and radiation necrosis after stereotactic radiosurgery of brain metastases: literature review. Cancer Radiother.

[16] Kano H, Kondziolka D, Lobato-Polo J, Zorro O, Flickinger JC, Lunsford LD (2010). T1/T2 matching to differentiate tumor growth from radiation effects after stereotactic radiosurgery. Neurosurgery.

[17] Leeman JE, Clump DA, Flickinger JC, Mintz AH, Burton SA, Heron DE (2013). Extent of perilesional edema differentiates radionecrosis from tumor recurrence following stereotactic radiosurgery for brain metastases. Neuro Oncol.

[18] Stockham AL, Tievsky AL, Koyfman SA, Reddy CA, Suh JH, Vogelbaum MA (2012). Conventional MRI does not reliably distinguish radiation necrosis from tumor recurrence after stereotactic radiosurgery. J Neurooncol.

[19] Matuszak J, Waissi W, Clavier J-B, Noël G, Namer I-J (2016). Métastases cérébrales: apport de l’acquisition tardive en TEP/TDM au 18F-FDG pour le diagnostic différentiel entre récurrence tumorale et radionécrose. Médecine Nucléaire.

[20] Zindler JD, Slotman BJ, Lagerwaard FJ (2014). Patterns of distant brain recurrences after radiosurgery alone for newly diagnosed brain metastases: implications for salvage therapy. Radiother Oncol.

[21] Ayala-Peacock DN, Peiffer AM, Lucas JT, Isom S, Kuremsky JG, Urbanic JJ (2014). A nomogram for predicting distant brain failure in patients treated with gamma knife stereotactic radiosurgery without whole brain radiotherapy. Neuro Oncol.

[22] Koiso T, Yamamoto M, Kawabe T, Watanabe S, Sato Y, Higuchi Y (2016). Follow-up results of brain metastasis patients undergoing repeat Gamma Knife radiosurgery. J Neurosurg.

[23] Kotecha R, Damico N, Miller JA, Suh JH, Murphy ES, Reddy CA (2017). Three or more courses of stereotactic radiosurgery for patients with multiply recurrent brain metastases. Neurosurgery.

[24] Shuto T, Fujino H, Inomori S, Nagano H (2004). Repeated gamma knife radiosurgery for multiple metastatic brain tumours. Acta Neurochir.

[25] Fritz C, Borsky K, Stark LS, Tanadini-Lang S, Kroeze SGC, Krayenbühl J (2018). Repeated courses of radiosurgery for new brain metastases to defer whole brain radiotherapy: feasibility and outcome with validation of the new prognostic metric brain metastasis velocity. Front Oncol.

[26] Shultz DB, Modlin LA, Jayachandran P, Von Eyben R, Gibbs IC, Choi CYH (2015). Repeat courses of stereotactic radiosurgery (SRS), deferring whole-brain irradiation, for new brain metastases after initial SRS. Int J Radiat Oncol Biol Phys.

[27] Jablonska PA, Serrano Tejero D, Calvo González A, Gimeno Morales M, Arbea Moreno L, Moreno-Jiménez M (2020). Repeated stereotactic radiosurgery for recurrent brain metastases: an effective strategy to control intracranial oligometastatic disease. Crit Rev Oncol Hematol.

[28] Nicosia L, Figlia V, Giaj-Levra N, Minniti G, Alongi F (2020). Repeated stereotactic radiosurgery for the treatment of relapsed brain metastases: is it time to give up whole-brain radiotherapy?. Oncoscience.

[29] Gaspar L, Scott C, Rotman M, Asbell S, Phillips T, Wasserman T (1997). Recursive partitioning analysis (RPA) of prognostic factors in three Radiation Therapy Oncology Group (RTOG) brain metastases trials. Int J Radiat Oncol Biol Phys.

[30] Gaspar LE, Scott C, Murray K, Curran W (2000). Validation of the RTOG recursive partitioning analysis (RPA) classification for brain metastases. Int J Radiat Oncol Biol Phys.

[31] Sperduto PW, Chao ST, Sneed PK, Luo X, Suh J, Roberge D (2010). Diagnosis-specific prognostic factors, indexes, and treatment outcomes for patients with newly diagnosed brain metastases: a multi-institutional analysis of 4,259 patients. Int J Radiat Oncol Biol Phys.

[32] Yamamoto M, Kawabe T, Higuchi Y, Sato Y, Nariai T, Watanabe S (2014). Validity of prognostic grading indices for brain metastasis patients undergoing repeat radiosurgery. World Neurosurg.

[33] Shibahara I, Kanamori M, Watanabe T, Utsunomiya A, Suzuki H, Saito R (2018). Clinical features of precocious, synchronous, and metachronous brain metastases and the role of tumor resection. World Neurosurg.

[34] Sun M, De Velasco G, Brastianos PK, Aizer AA, Martin A, Moreira R (2019). The development of brain metastases in patients with renal cell carcinoma: epidemiologic trends, survival, and clinical risk factors using a population-based cohort. Eur Urol Focus.

[35] Shindorf ML, Jafferji MS, Goff SL (2020). Incidence of asymptomatic brain metastases in metastatic colorectal cancer. Clin Colorectal Cancer.

[36] Jiang X (2020). Identification of patients with brain metastases with favorable prognosis after local and distant recurrence following stereotactic radiosurgery. Cancer Manag Res.

[37] Davies MA, Saiag P, Robert C, Grob J-J, Flaherty KT, Arance A (2017). Dabrafenib plus trametinib in patients with BRAFV600-mutant melanoma brain metastases (COMBI-MB): a multicentre, multicohort, open-label, phase 2 trial. Lancet Oncol.

[38] Tawbi HA, Forsyth PA, Algazi A, Hamid O, Hodi FS, Moschos SJ (2018). Combined nivolumab and ipilimumab in melanoma metastatic to the brain. N Engl J Med.

[39] Medikonda R, Srivastava S, Kim T, Xia Y, Kim J, Jackson C (2021). Development of new brain metastases in triple negative breast cancer. J Neurooncol.

[40] Murthy RK, Loi S, Okines A, Paplomata E, Hamilton E, Hurvitz SA (2020). Tucatinib, trastuzumab, and capecitabine for HER2-positive metastatic breast cancer. N Engl J Med.

[41] Veccia A, Kinspergher S, Dipasquale M, Caffo O (2021). Management of brain metastases from lung cancer in the era of immunotherapy: a review of the literature. Future Oncol.

[42] Lauko A, Thapa B, Venur VA, Ahluwalia MS (2018). Management of brain metastases in the new era of checkpoint inhibition. Curr Neurol Neurosci Rep.

